# Basic Fibroblast Growth Factor Fused with Tandem Collagen-Binding Domains from* Clostridium histolyticum *Collagenase ColG Increases Bone Formation

**DOI:** 10.1155/2018/8393194

**Published:** 2018-03-25

**Authors:** Hiroyuki Sekiguchi, Kentaro Uchida, Osamu Matsushita, Gen Inoue, Nozomu Nishi, Ryo Masuda, Nana Hamamoto, Takaki Koide, Shintaro Shoji, Masashi Takaso

**Affiliations:** ^1^Department of Orthopedic Surgery, Kitasato University School of Medicine, 1-15-1 Minami-ku, Kitasato, Sagamihara City, Kanagawa, Japan; ^2^Department of Bacteriology, Okayama University Graduate School of Medicine, Dentistry and Pharmaceutical Sciences, 2-5-1 Kita-ku Shikata-cho, Okayama, Japan; ^3^Life Science Research Center, Kagawa University, 1750-1 Kita-gun Miki-cho, Kagawa, Japan; ^4^Department of Chemistry and Biochemistry, School of Advanced Science and Engineering, Waseda University, 3-4-1 Okubo, Shinjuku-ku, Tokyo, Japan; ^5^Okayama University Medical School, 2-5-1 Shikata-cho, Kita-ku, Okayama, Japan

## Abstract

Basic fibroblast growth factor 2 (bFGF) accelerates bone formation during fracture healing. Because the efficacy of bFGF decreases rapidly following its diffusion from fracture sites, however, repeated dosing is required to ensure a sustained therapeutic effect. We previously developed a fusion protein comprising bFGF, a polycystic kidney disease domain (PKD; s2b), and collagen-binding domain (CBD; s3) sourced from the* Clostridium histolyticum* class II collagenase, ColH, and reported that the combination of this fusion protein with a collagen-like peptide, poly(Pro-Hyp-Gly)_10_, induced mesenchymal cell proliferation and callus formation at fracture sites. In addition,* C. histolyticum* produces class I collagenase (ColG) with tandem CBDs (s3a and s3b) at the C-terminus. We therefore hypothesized that a bFGF fusion protein containing ColG-derived tandem CBDs (s3a and s3b) would show enhanced collagen-binding activity, leading to improved bone formation. Here, we examined the binding affinity of four collagen anchors derived from the two clostridial collagenases to H-Gly-Pro-Arg-Gly-(Pro-Hyp-Gly)_12_-NH_2_, a collagenous peptide, by surface plasmon resonance and found that tandem CBDs (s3a-s3b) have the highest affinity for the collagenous peptide. We also constructed four fusion proteins consisting of bFGF and s3 (bFGF-s3), s2b-s3b (bFGF-s2b-s3), s3b (bFGF-s3b), and s3a-s3b (bFGF-s3a-s3b) and compared their biological activities to those of a previous fusion construct (bFGF-s2b-s3) using a cell proliferation assay in vitro and a mouse femoral fracture model in vivo. Among these CB-bFGFs, bFGF-s3a-s3b showed the highest capacity to induce mesenchymal cell proliferation and callus formation in the mice fracture model. The poly(Pro-Hyp-Gly)_10_/bFGF-s3a-s3b construct may therefore have the potential to promote bone formation in clinical settings.

## 1. Introduction

Basic fibroblast growth factor (bFGF), a mitogenic protein with angiogenic properties, is involved in bone remodeling during early bone repair [1, 2]. Recombinant human bFGF has demonstrated efficacy in the regeneration of bone fractures and defects in animal models of osteoporosis [3, 4]. In two recent clinical trials, bFGF treatment accelerated bone union at osteotomy and tibial fracture sites [5, 6]. Although these findings strongly indicate that bFGF promotes bone remodeling and regeneration, exogenously added bFGF is rapidly diffused from bone defect sites and can induce side effects such as tumor activation, kidney toxicity, and thrombocytopenia [7, 8]. Acceleration of bone formation in clinical settings therefore requires a means to ensure bFGF retention at the site of fracture.

A previous study aimed to increase the efficacy of bFGF by adding collagen-binding domains from mammalian collagenase, von Willebrand factor, and* Clostridium collagenase* to bFGF to enhance its collagen-binding ability [9–12].* Clostridium histolyticum*, a pathogenic bacterium of gas gangrene, secretes two classes of collagenase (class I, ColG; class II, ColH). These enzymes commonly contain catalytic (s1), polycystic kidney disease (PKD, s2), and collagen-binding domains (CBD, s3). However, copy numbers of PKD and CBD in the C-terminal collagen anchors differ between ColG (s2-s3a-s3b) and ColH (s2a-s2b-s3) [13, 14]. We previously demonstrated that fusion proteins consisting of bFGF and either CBD (bFGF-s3) or PKD-CBD (bFGF-s2b-s3) derived from ColH accelerated bone formation in rat femoral bone compared to native bFGF when applied to collagen sheets [15]. Further, when combined with high-density collagen sheets, bFGF-s2b-s3 had long retention times and promoted more bone formation than bFGF-s3 [12]. In mouse bone fracture models, the combination of bFGF-s2b-s3 with the collagen-like peptide poly(Pro-Hyp-Gly)_10_ also promoted more callus (newly formed bone during fracture healing) formation compared to bFGF [16]. In a more recent study, a fusion protein comprising galectin-9 and tandem CBDs (s3a and s3b) sourced from ColG showed higher binding activity than galectin-9 fused with ColH-derived PKD and CBD (s2b and s3) [17]. We therefore hypothesized that a bFGF fusion protein with ColG-derived tandem CBDs would show enhanced collagen-binding activity, leading to improved bone formation.

Here, we evaluated the dissociation constants of various collagen anchors and minicollagen* in vitro*. We then constructed fusion proteins consisting of bFGF and either single (bFGF-s3b) or tandem CBD(s) (bFGF-s3a-s3b) derived from ColG and compared their function in bone formation with that of two prior fusion proteins consisting of bFGF and collagen anchors derived from ColH.

## 2. Materials and Methods

### 2.1. A Minicollagen Peptide and a Collagen-Like Polypeptide

A minicollagen peptide, H-Gly-Pro-Arg-Gly-(Pro-Hyp-Gly)_12_-NH_2_, was synthesized using a 9-fluorenylmethoxycarbonyl- (Fmoc-) based strategy on Rink-amide resins (Novabiochem, San Diego, CA). In each cycle, Fmoc-amino acids (5 equivalents; Novabiochem) were reacted in the presence of* N*,*N*′-diisopropylcarbodiimide (5 equivalents; Wako Pure Chemical, Osaka, Japan) and 1-hydroxybenzotriazole (5 equivalents; Wako Pure Chemical) in* N*,*N*-dimethylformamide for 90 minutes. Fmoc deprotection was conducted using 20% (v/v) piperidine in DMF for 20 minutes. Peptide cleavage and deprotection steps consisted of treatment with a standard trifluoroacetic acid (TFA) scavenger cocktail (TFA :* m*-cresol : thioanisole : water : ethanedithiol = 82.5 : 5 : 5 : 5 : 2.5, v/v) for 4 hours at room temperature. The peptide was purified by HPLC using a Cosmosil 5C18-AR-II column (20 × 250 mm, Nacalai Tesque, Kyoto, Japan) with CH_3_CN in water, both containing 0.05% (v/v) TFA. Product purity was confirmed by RP-HPLC using a Cosmosil 5C18-AR-II column (4.6 × 250 mm, Nacalai Tesque) with a linear gradient of CH_3_CN in water, both containing 0.05% (v/v) TFA. Mass spectrometric analysis was conducted using a Bruker Autoflex III MALDI-TOF MS (Bruker Daltonics, Leipzig, Germany), H-Gly-Pro-Arg-Gly-(Pro-Hyp-Gly)_12_-NH_2_: MS (MALDI-TOF)* m/z* calculated for C_159_H_232_N_44_O_52_ ([M + H]^+^), 3590.7; found, 3590.6 (Supplementary Figure 1). Circular dichroism analysis of the peptide was conducted according to a previous report (Supplementary Figure 2) [18]. The melting temperature for the triple helix of the peptide in H_2_O was estimated to be 76.2°C. The collagen-like polypeptide poly(Pro-Hyp-Gly)_10_ was obtained from PHG Co., Ltd. (Hyogo, Japan) [19]. The material properties of poly(Pro-Hyp-Gly)_10_, such as heat resistance and particle size, have been detailed elsewhere [16].

### 2.2. Collagen Anchors Derived from the Clostridial Collagenases ColG and ColH

CBD (s3) and PKD-CBD (s2b-s3), sourced from the class II collagenase of* C. histolyticum*, ColH, were purified as described previously [20]. CBD (s3b) and CBD-CBD (s3a-s3b), sourced from the class I collagenase of* C. histolyticum*, ColG, were purified as described previously [21].

### 2.3. Quantitative Analysis of the Binding of a Collagen Anchor to a Collagenous Peptide

The binding of collagen anchors to the minicollagen peptide was assessed by surface plasmon resonance using a BIACORE apparatus (Biacore, Uppsala, Sweden) as reported previously [22]. Briefly, the peptide was dissolved in 10 mM sodium acetate (pH 6.0) at a concentration of 0.1 mg/ml and covalently immobilized on a CM5 sensor chip (Biacore) using the standard amine coupling procedure recommended by the manufacturer. Resonance in 10 mM sodium HEPES (pH 7.4), 150 mM NaCl, 1 mM CaCl_2_, and 0.005% Tween-20 was determined at a flow rate of 20 *μ*l/min at 25°C. Following each binding step, the chip was regenerated using a 180 s pulse of 0.1 M HCl. The apparent dissociation constant, *K*_D_ (app), was determined using equilibrium binding data for eight protein concentrations (100 nM–300 *μ*M) by direct fitting to the following equation by the least squares method:

(1) $$\begin{array}{c} \text{cRU}={\text{cRU}}_{\max}\times \frac{\left[\text{protein}\right]}{\left(K_{\text{D}}+\left[\text{protein}\right]\right)}, \end{array}$$

where cRU indicates the response at equilibrium corrected for bulk refractive index errors by blocking of a sham-coupled flow cell with ethanolamine, [protein] indicates the analyte concentration, and *K*_D_ indicates the dissociation constant.

### 2.4. Collagen-Binding bFGF

**Figure 1:**
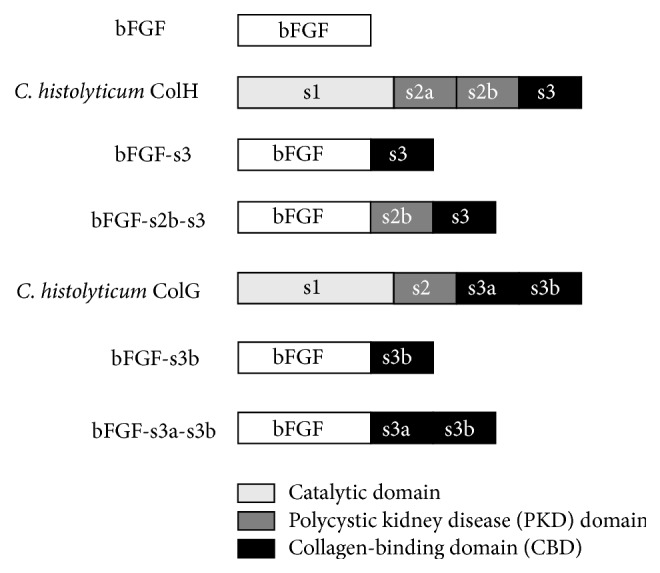
Structures of the four collagen-binding-basic fibroblast growth factor constructs.

**Figure 2:**
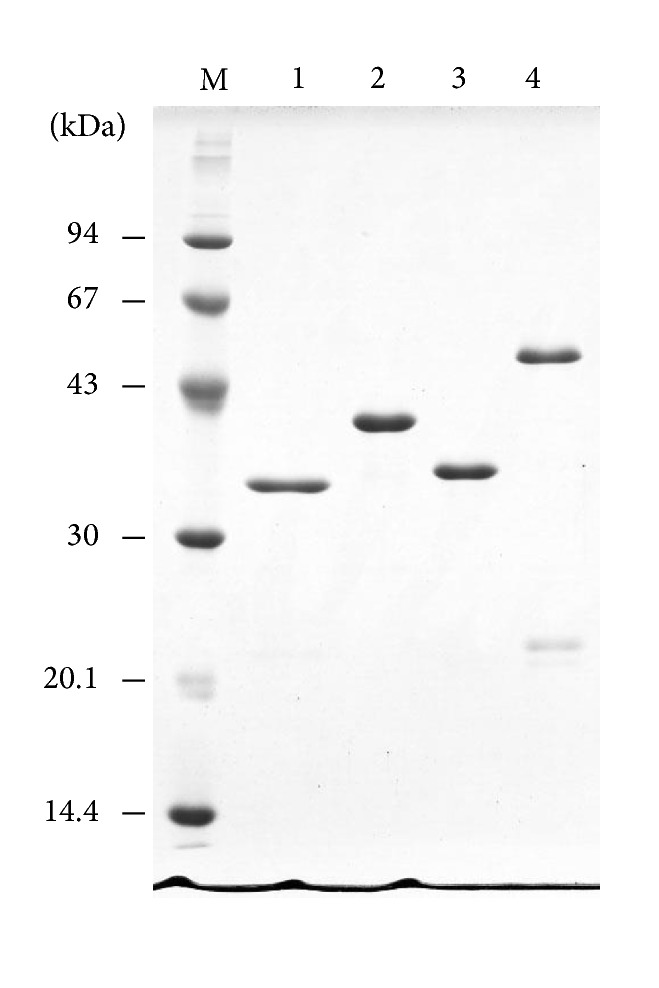
SDS-polyacrylamide gel showing the molecular masses of the various collagen-binding bFGF proteins. GST-fusion proteins of collagen-binding bFGF (CB-bFGFs) were produced using pGEX-4T vectors. The proteins were purified by glutathione affinity chromatography, and the GST moiety was cleaved off using thrombin proteinase. CB-bFGFs were isolated using a Heparin-Sepharose column and the proteins (1 *μ*g each) were analyzed by SDS-polyacrylamide gel electrophoresis (15% gel). M, molecular mass marker; 1, bFGF-s3 (calculated mass, 30,920.37); 2, bFGF-s2b-s3 (calculated mass, 41,516.07); 3, bFGF-s3b (calculated mass, 30,925.34); and 4, bFGF-s3a-s3b (calculated mass, 44,193.58). Numbers on the left indicate molecular mass (kDa) according to the molecular marker.

Four collagen-binding bFGF fusion proteins (CB-bFGFs) were used in this study (Figures 1 and 2). Two fusion proteins, bFGF-s3 and bFGF-s2b-s3, consisting of human bFGF and CBD or PKD-CBD derived from ColH, were constructed as previously described [10, 12]. To construct bFGF-s3b, an expression plasmid, pCHG115, encoding a fusion protein between GST and a C-terminal CBD (s3b, ColG) [21] was digested with* Bam*HI and* Eco*RI at the linker region and ligated with a hbFGF-encoding DNA fragment using* Bgl*II and* Eco*RI linkers. The ligation mixture was transformed into* E. coli *DH5*α* cells and the nucleotide sequence of the resulting plasmid (pCHG115-hbFGF) was confirmed by Sanger sequencing. The plasmid was transformed into* E. coli* BL21 CodonPlus RIL (Agilent Technologies, Santa Clara, CA) to express the GST-bFGF-s3b fusion protein. The protein was purified by glutathione affinity chromatography (GE Healthcare), the GST moiety was cleaved off using thrombin protease (GE Healthcare), and bFGF-s3b was further purified using an Heparin-Sepharose (GE Healthcare) affinity column as described previously [12]. Another fusion protein comprising bFGF and tandem CBDs derived from ColG (bFGF-s3a-s3b) was produced in the same manner using the pCHG112 expression plasmid [21].

### 2.5. Proliferation Assay

The periosteum of distal femurs was harvested from 10-week-old male Wistar rats [12] and digested with 0.2% type I collagenase (Wako Pure Chemical) for 2 hours at 37°C. The digested sample was passed through a sterile 40 *μ*m filter to extract single-cell suspensions of periosteal mesenchymal cells (PMCs), which were seeded at 1 × 10^4^ cells/cm^2^ in 6-well plates containing *α*-minimum essential medium supplemented with 10% fetal bovine serum. The PMCs were cultured for 7 days, detached by treating with 0.25% trypsin–EDTA solution for 3 minutes, and 1.25 × 10^3^ cells/well were cultured in 96-well plates (BD Falcon, NJ, USA). *α*-MEM containing poly(Pro-Hyp-Gly)_10_ with bFGF, bFGF-s3, bFGF-s2b-s3, bFGF-s3b, or bFGF-s3a-s3b was added to the cultured cells at concentrations of 0.1, 1, and 10 pM. After two days of treatment, cell proliferation was evaluated by the tetrazolium salt method using a WST-8 kit (Nacalai Tesque, Tokyo, Japan) [15]. Results were calculated by dividing the cell number of CB-bFGFs-stimulated groups by mean cell number of the culture medium (*α*-MEM without bFGF) stimulated group (control) and are expressed as fold increase.

### 2.6. Fracture Model

A femur fracture model was generated using 9-week-old C57BL/6J mice. The mice were fed a standard laboratory diet, CRF-1 (Oriental Yeast, Tokyo, Japan), and housed under controlled temperature (23 ± 2°C) and humidity (55 ± 10%) conditions and a 12-hour light/dark cycle. For femur fractures, a 4 mm left medial parapatellar incision was made under sterile conditions and a 0.5 mm hole was drilled in the intracondylar notch. A 0.2 mm tungsten guide wire was retrogradely inserted into the intramedullary canal, and a section of the femur was removed using a 0.22 mm diameter wire saw with a lateral approach. After withdrawing the guide wire, the fracture was stabilized by insertion of a 0.5 mm diameter stainless steel screw into the intramedullary canal. Immediately following creation of the fracture, PBS (control) or poly(Pro-Hyp-Gly)_10_ gel containing bFGF, bFGF-s3, bFGF-s2b-s3, bFGF-s3b, or bFGF-s3a-s3b was injected into the fracture site (*n* = 8, per treatment group). The bFGF dose used was based on the results of a previous study [16]. To generate the CB-bFGFs/poly(Pro-Hyp-Gly)_10_ construct, 5 *μ*l of 11.6 *μ*M CB-bFGFs solution was mixed with 20 *μ*l of 1% poly(Pro-Hyp-Gly)_10_ gel to make a 25 *μ*l mixture containing 58 pmoles of the fusion protein (final concentration, 2.32 *μ*M). All animal procedures met the guidelines of the Animal Ethics Committee of Kitasato University.

### 2.7. Quantification of Callus Volume and Bone Mineral Content

Four weeks after the fracture surgery, femurs were removed from sacrificed mice and fixed in 4% paraformaldehyde solution for two days at 4°C. The femurs were transferred to PBS and imaged using an inspeXio SMX-90CT microfocus X-ray CT system (Shimadzu, Tokyo, Japan) under the following conditions: acceleration voltage, 90 kV; current, 110 mA; voxel size, 20 lm/pixel; and matrix size, 1024 × 1024. Callus volume (CV) and bone mineral content (BMC) were measured from 10 mm regions (500 slices) from the mid-femur in micro-CT images using Tri-3D-Bon three-dimensional (3D) image analysis software (Ratoc System Engineering, Tokyo, Japan), as previously described [20, 23]. BMC was estimated by comparing the measured densities of each femur sample to those determined from a hydroxyapatite (HA) calibration curve constructed from phantom image data obtained at 0.2, 0.3, 0.4, 0.5, 0.6, 0.7, and 0.8 g HA/cm^3^. CV was indicated by a threshold density value ≥ 0.3 g/cm^3^.

### 2.8. Histological Evaluation

To examine the effects of poly(Pro-Hyp-Gly)_10_/bFGF-s3a-s3b treatment on bone formation, femurs were harvested from control and treated mice two weeks after the fracture surgeries and demineralized in a 0.5 M EDTA solution for two weeks. The remaining tissue was embedded in paraffin and 3 *μ*m femoral sections were generated using a microtome. Histological analysis was performed on hematoxylin/eosin- (HE-) stained sections.

### 2.9. Statistical Analysis

Differences among the control, bFGF, bFGF-s3, bFGF-s2b-s3, bFGF-s3b, and bFGF-s3a-s3b groups were assessed using one-way ANOVA with Fisher's least significant difference (LSD) test. A significant difference was defined by *p* < 0.05. All statistical analyses were conducted using SPSS software (Version 19.0; SPSS Inc., Chicago, IL).

## 3. Results

### 3.1. Binding Affinity of Collagen Anchors

Dissociation constants of four collagen anchors to the minicollagen peptide, H-Gly-Pro-Arg-Gly-(Pro-Hyp-Gly)_12_-NH_2_, were determined by surface plasmon resonance (Table 1). Although s2b-s3 (PKD-CBD, ColH) had a lower *Kd* value than s3 alone (CBD, ColH), s3b (CBD, ColG) and s3a-s3b (CBD-CBD, ColG) had *Kd* values that were approximately 10-fold lower than those of both ColH anchors. Therefore, the ColG-derived anchors bind more tightly to the collagenous peptide than the ColH-derived anchors.

### 3.2. *In Vitro* Biological Activities of Fusion Proteins

**Figure 3:**
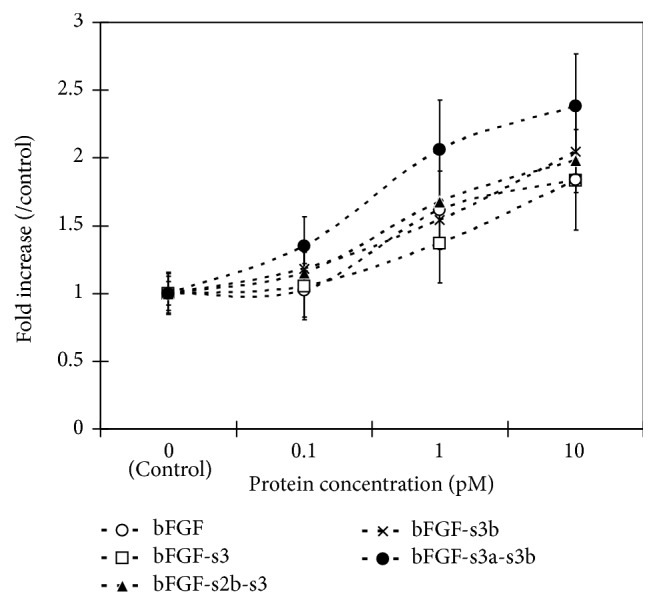
In vitro proliferation activity of poly(Pro-Hyp-Gly)_10_/bFGF and poly(Pro-Hyp-Gly)_10_/CB-bFGFs. Cultured periosteal mesenchymal cells stimulated with poly(Pro-Hyp-Gly)_10_/bFGF, poly(Pro-Hyp-Gly)_10_/bFGF-s3, poly(Pro-Hyp-Gly)_10_/bFGF-s2b-s3, poly(Pro-Hyp-Gly)_10_/bFGF-s3b, or poly(Pro-Hyp-Gly)_10_/bFGF-s3a-s3b. The constructs induced periosteal mesenchymal cell proliferation in a dose-dependent manner. Cell numbers were quantified two days after treatment. Data are presented as the mean ± SE (*n* = 8).

The biological activities of the poly(Pro-Hyp-Gly)_10_/CB-bFGFs were evaluated by measuring the proliferation of rat PMCs* in vitro* (Figure 3). Two days after treatment with 0.1 pM bFGF-s3a-s3b, the number of cultured PMCs had significantly increased compared to the control (*α*-MEM) treatment group (Figure 3, *p* < 0.05). In contrast, no significant increases were detected in bFGF-, bFGF-s3-, bFGF-s2b-s3-, or bFGF-s3b-treated cells. In addition, 1 pM bFGF-s3a-s3b significantly increased the number of cultured PMCs compared to control, bFGF, bFGF-s3, bFGF-s2b-s3, and bFGF-s3b. 10 pM bFGF-s3a-s3b significantly increased the number of cultured PMCs compared to control, bFGF, and bFGF-s3. When concentrations of bFGF or CB-bFGFs were increased to 1 or 10 pM, cell numbers were significantly increased for all growth factor-treated cells compared to *α*-MEM-treated cells.

### 3.3. Callus Formation by CB-bFGF/Poly(Pro-Hyp-Gly)_10_ Composite* In Vivo*

**Figure 4:**
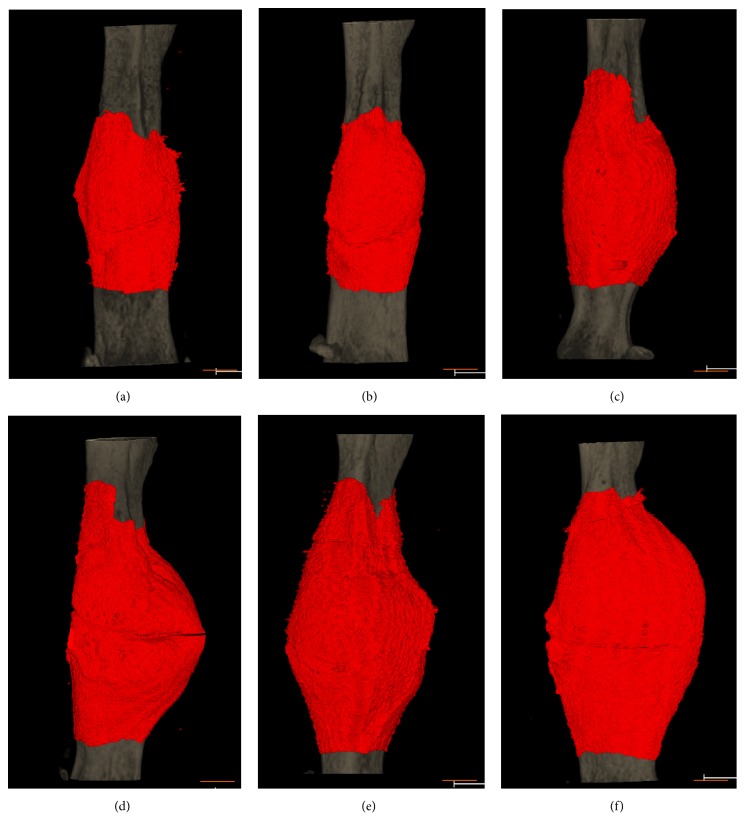
3D micro-CT images of femurs after injection of poly(Pro-Hyp-Gly)_10_ loaded with bFGF and CB-bFGFs. Three-dimensional images were created from 10 mm regions (500 slices) from the mid-femur using Tri-3D-Bon 3D image analysis software. 3D micro-CT images of fractured mouse femurs treated with (a) PBS, (b) bFGF, (c) bFGF-s3, (d) bFGF-s2b-s3, (e) bFGF-s3b, and (f) bFGF-s3a-s3b after four weeks of recovery. Red: callus; gray: existing bone. The scale bars indicate 3 mm.

**Figure 5:**
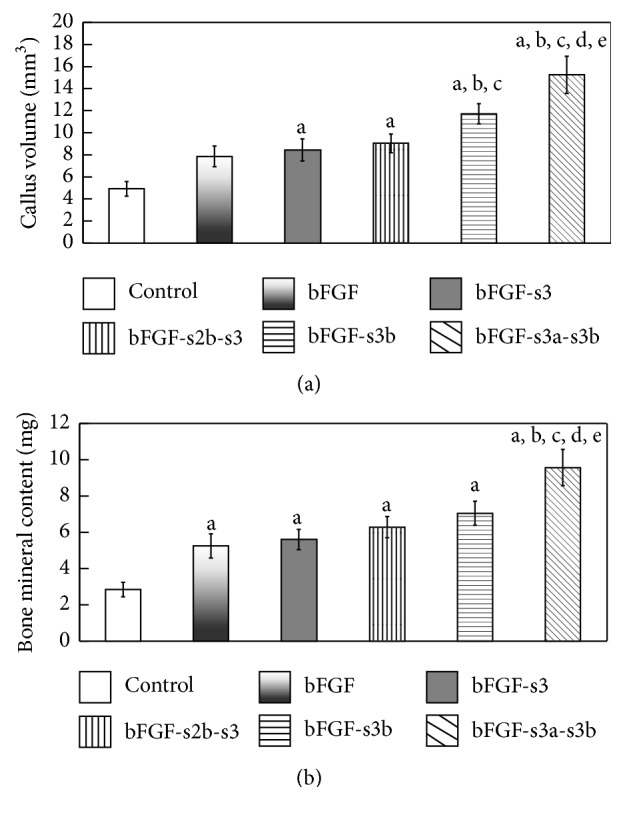
Analysis of 3D micro-CT images of mouse femurs four weeks after injection of poly(Pro-Hyp-Gly)_10_ loaded with bFGF and CB-bFGFs into femoral fracture sites. We analyzed 3D images created from 10 mm regions (500 slices) from the mid-femur using Tri-3D-Bon 3D image analysis software four weeks after creation of the fracture. (a) Callus volume (mm^3^) and (b) bone mineral content (mg). Data are presented as the mean ± SE (*n* = 8). ^a^*p* < 0.05 compared with the control group. ^b^*p* < 0.05 compared with the bFGF group. ^c^*p* < 0.05 compared with the bFGF-s3 group. ^d^*p* < 0.05 compared with the bFGF-s2b-s3 group. ^e^*p* < 0.05 compared with the bFGF-s3b group.

Poly(Pro-Hyp-Gly)_10_ gel was mixed with either PBS, bFGF (controls), or one of the four prepared CB-bFGFs and applied to the femur fracture site of mice. After four weeks of recovery, callus formation at the fracture sites was assessed by micro-CT image analysis (Figure 4). CV and BMC were significantly higher in mice treated with poly(Pro-Hyp-Gly)_10_ in combination with bFGF or the CB-bFGF fusion proteins (Figure 5, *p* < 0.05) than in those treated with PBS. Notably, however, bFGF-s3b- and bFGF-s3a-s3b-treated factures had higher CV compared to those treated with bFGF (Figure 5, *p* < 0.05). Among the four fusion proteins, bFGF-s3a-s3b exhibited the highest efficacy for bone repair, with CV and BMC being significantly higher than those of all the other groups (Figure 5, *p* < 0.05).

### 3.4. Histomorphometric Findings

**Figure 6:**
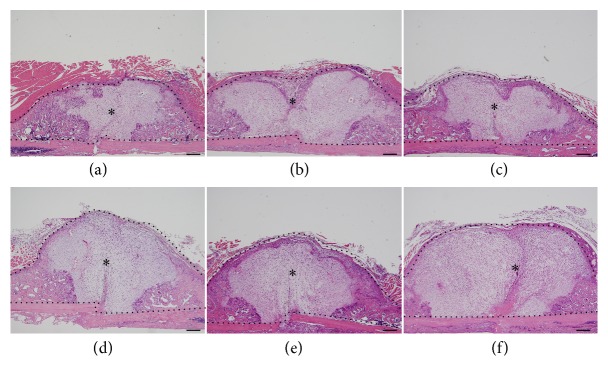
Representative hematoxylin and eosin-stained tissue sections at 2 weeks of fracture healing. Histological images of fractured mouse femurs treated with (a) PBS, (b) bFGF, (c) bFGF-s3, (d) bFGF-s2b-s3, (e) bFGF-s3b, and (f) bFGF-s3a-s3b after two weeks of recovery. Regions enclosed by black dotted lines (*∗*) in (a)–(f) indicate callused areas. Scale bar = 500 *μ*m.

To investigate the mechanisms by which poly(Pro-Hyp-Gly)_10_/bFGF-s3a-s3b enhances new bone formation, treated fracture sites were examined by histology two weeks after surgical fracture induction, when soft callus formation was first detected in the mouse femoral fracture model. Compared to controls, the fracture sites of mice treated with either bFGF-s2b-s3, bFGF-s3b, or bFGF-s3a-s3b showed larger calluses with fibroblastic and chondrocytic cells (Figure 6). Notably, calluses formed by treatment with bFGF-s3a-s3b were clearly larger than those observed under the other treatments (Figure 6), indicating that poly(Pro-Hyp-Gly)_10_/bFGF-s3a-s3b accelerates periosteal cell proliferation and chondrogenesis.

## 4. Discussion

Clostridial collagenases possess collagen anchors at their C-termini. These anchors bind to collagen fibrils and collagenous peptides with a triple-helical conformation, but not to single-chain collagen polypeptides [21]. The anchors are made of two types of domain, PKD and CBD, with the former enhancing the binding of the latter. Further, the enzymes possess collagen anchors made of various copy numbers of PKD(s) and CBD(s). We previously reported the callus-inducing potential of composite materials containing a collagen carrier (high-density collagen sheet/powder or demineralized bone matrix) combined with bFGF fused with an anchor comprising a single copy each of PKD and CBD (bFGF-s2b-s3) derived from the* Clostridium histolyticum* class II collagenase, ColH [20, 23]. Recently, we evaluated the use of a collagenous peptide gel instead of a collagen carrier, since the former is more easily injected. In aged mouse fracture models, a composite material containing bFGF-s2b-s3 and poly(Pro-Hyp-Gly)_10_ stimulated greater callus formation than one made of bFGF and poly(Pro-Hyp-Gly)_10_ [16]. Hence, we speculated that the efficacy of this composite material could be optimized further by substituting anchors with various binding affinities to the collagenous peptide carrier.

To estimate the binding affinity of various anchors to the peptide carrier, poly(Pro-Hyp-Gly)_10_, we synthesized a longer collagenous peptide, H-Gly-Pro-Arg-Gly-(Pro-Hyp-Gly)_12_-NH_2_, as a ligand for surface plasmon resonance assay. The *K*_D_ value of a single CBD (s3b) to this peptide was 4.54 ± 0.15 × 10^−5^ M, which is similar to that reported previously to a shorter collagenous peptide, (G(POG)_8_) (5.72 ± 0.473 × 10^−5^ M) [22], indicating that the quantitative analysis performed here is reproducible. Collagen anchors (s3a-s3b and s3b) derived from the class I enzyme (ColG) had significantly higher affinity to this peptide than anchors (s2b-s3 and s3) derived from the class II enzyme (ColH), suggesting that the former are more appropriate as peptide carriers than the latter. The presence of an additional CBD (s3a) did not significantly enhance binding of ColG s3b to the synthetic peptide, suggesting that the peptide used for this assay may still be too short to allow simultaneous binding of the two CBDs. Alternatively, binding between s3a and the collagen peptide may be too weak to be reflected in the apparent *K*_D_ value. A binding assay and/or small-angle X-ray diffraction study using a longer collagenous peptide would be necessary to solve this.

Provided that efficacy correlates with binding affinity, greater callus formation may be expected by fusing bFGF with ColG anchors and combination with the collagenous peptide. Hence, we prepared four CB-bFGFs with one of the anchors described above. To confirm that the bFGF moiety in each CB-bFGF construct was intact, cell proliferation assays were performed* in vitro.* The four CB-bFGFs and bFGF promoted PMC proliferation in a dose-dependent manner, indicating that the bFGF moiety is active despite the various anchor moiety combinations. The activity of poly(Pro-Hyp-Gly)_10_/bFGF-s3a-s3b was higher than that of the other poly(Pro-Hyp-Gly)_10_/CB-bFGFs at lower concentrations (0.1–1.0 pM), which might be due to the binding of bFGF-s3a-s3b to poly(Pro-Hyp-Gly)_10_.

We also compared the osteogenic potential of composite materials made of poly(Pro-Hyp-Gly)_10_ and either of the four CB-bFGFs using a mouse femur fracture model. The binding affinity of collagen anchors may affect the osteogenic activity of the corresponding fusion proteins, given that the rapid diffusion of target molecules from defect sites would limit their osteogenic potential [12]. We previously demonstrated that bFGF-PKD-CBD (bFGF-s2b-s3) has higher binding affinity to a collagen carrier and induces greater bone formation than bFGF-CBD (bFGF-s3) [12]. Among the collagen anchors used in the present study, ColG CBD(s) showed approximately 10-fold higher affinity than ColH anchors to a collagenous peptide. This is consistent with our* in vivo* findings that treatment with bFGF fused with ColG anchors combined with a collagenous peptide carrier more efficiently accelerated callus formation than treatment with bFGF fused with ColH anchors. Therefore, osteogenic potential is correlated with the binding affinity of collagen anchors. Further, tandem CBDs (ColG) are better for increasing the retention time of bFGF at fracture sites, thereby accelerating callus formation, when introduced together with poly(Pro-Hyp-Gly)_10_. These results suggest that materials comprising bFGF-s3a-s3b and poly(Pro-Hyp-Gly)_10_ may constitute promising therapies for stimulating fracture healing in clinical settings.

bFGF expressed in periosteum with fibroblastic and chondrocytic cells during the early stages of fracture healing and exogenously added bFGF stimulate mesenchymal cell proliferation and chondrogenesis in fracture sites [2, 24]. Histological analysis in the early stage of the fracture healing process at 2 weeks showed that bFGF-s3a-s3b induced greater soft callus formation with fibroblastic and chondrocytic cells. This larger callus formation by the bFGF-s3a-s3b/poly(Pro-Hyp-Gly)_10_ construct may be due to the acceleration of mesenchymal proliferation and chondrogenesis in the early stage of fracture healing.

Several limitations of this study warrant mention. First, while we have demonstrated the therapeutic potential of bFGF-s3a-s3b/poly(Pro-Hyp-Gly)_10_ for treating fractures, the mechanisms underlying their promotion of bone formation remain to be determined. Second, it remains to be determined whether the retention time of bFGF correlates with the binding constant of the CBD to the collagen peptides used. Finally, the retention time of bFGF after the injection of bFGF-s3a-s3b/poly(Pro-Hyp-Gly)_10_ into fracture sites remains to be determined. Further in vitro and in vivo investigation of the half-life of bFGF-s3a-s3b/poly(Pro-Hyp-Gly)_10_ will reveal the mechanisms underlying these bone formation-promoting effects.

## 5. Conclusion

The combination of a recombinant collagen-binding bFGF fusion protein containing tandem CBDs from the* C. histolyticum* class I collagenase, ColG, and the collagen-like peptide, poly(Pro-Hyp-Gly)_10_, induced significant callus formation when injected into mouse femoral fracture sites. The high osteogenic properties of bFGF-s3a-s3b/poly(Pro-Hyp-Gly)_10_ suggest that this composite material has the potential to promote fracture healing in clinical settings.

## Figures and Tables

**Table 1:** Binding affinity of various collagen anchors.

Collagen anchor	*K*_D_ (×10^−5^ M)
s3	75.2 ± 0.41
s2b-s3	44.5 ± 0.55
s3b	4.54 ± 0.15
s3a-s3b	4.46 ± 0.45

Anchor proteins were dissolved in HBS-Ca buffer at concentrations ranging from 1 × 10^−7^ M to 3 × 10^−4^ M. Binding to the collagenous peptide, H-Gly-Pro-Arg-Gly-(Pro-Hyp-Gly)_12_-NH_2_, was measured by surface plasmon resonance. Data were directly fitted to the equation described in the Materials and Methods by the least squares method to calculate the apparent dissociation constant (*K*_D_) and uncertainty values.

## Supplementary Materials

Supplementary Materials Supplementary Figure  1: mass spectrometric analysis of H-Gly-Pro-Arg-Gly-(Pro-Hyp-Gly)_12_-NH_2_ was conducted using a Bruker Autoflex III MALDI-TOF MS (Bruker Daltonics, Leipzig, Germany). H-Gly-Pro-Arg-Gly-(Pro-Hyp-Gly)_12_-NH_2_: MS (MALDI-TOF)* m/z* calculated for C_159_H_232_N_44_O_52_ ([M + H]^+^), 3590.7; found, 3590.6 (Supplementary Figure  1). Supplementary Figure  2: CD spectral analysis of H-Gly-Pro-Arg-Gly-(Pro-Hyp-Gly)_12_-NH_2_ showed the presence of a positive signal around 220 nm (Supplementary Figure  2(a)). H-Gly-Pro-Arg-Gly-(Pro-Hyp-Gly)_12_-NH_2_ retained the positive peak around 220 nm, even when treated at 65°C (Supplementary Figure  2(b))
