# Supplementary material for: Basic Fibroblast Growth Factor Fused with Tandem Collagen-Binding Domains from Clostridium histolyticum Collagenase ColG Increases Bone Formation
Source: Biomed Res Int. 2018 Mar 25;2018:8393194. doi: 10.1155/2018/8393194 (PMC5889866; doi:10.1155/2018/8393194)
Supplement: Supplementary Materials — Supplementary Figure 1: mass spectrometric analysis of H-Gly-Pro-Arg-Gly-(Pro-Hyp-Gly)12-NH2 was conducted using a Bruker Autoflex III MALDI-TOF MS (Bruker Daltonics, Leipzig, Germany). H-Gly-Pro-Arg-Gly-(Pro-Hyp-Gly)12-NH2: MS (MALDI-TOF) m/z calculated for C159H232N44O52 ([M + H]+), 3590.7; found, 3590.6 (Supplementary Figure 1). Supplementary Figure 2: CD spectral analysis of H-Gly-Pro-Arg-Gly-(Pro-Hyp-Gly)12-NH2 showed the presence of a positive signal around 220 nm (Supplementary Figure 2(a)). H-Gly-Pro-Arg-Gly-(Pro-Hyp-Gly)12-NH2 retained the positive peak around 220 nm, even when treated at 65°C (Supplementary Figure 2(b)) [file 8393194.f1.docx]

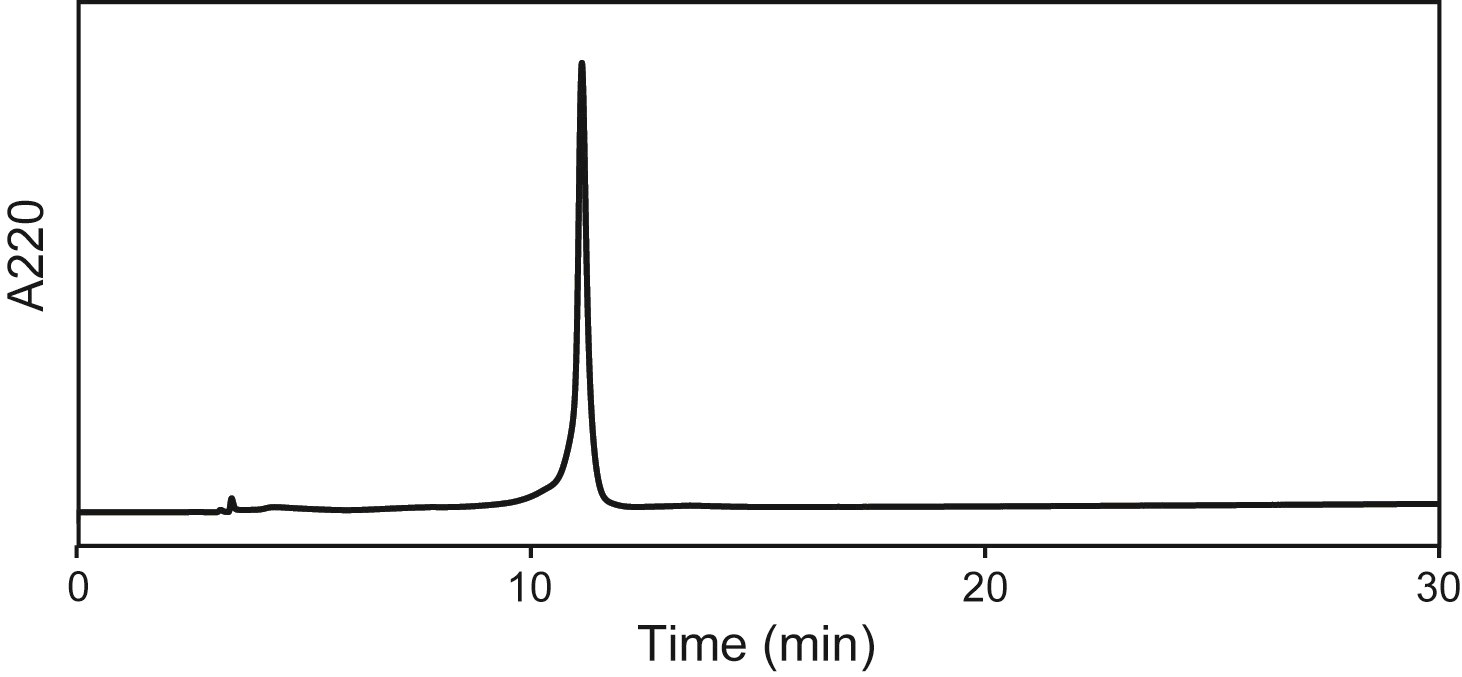


**Supplementary Fig. 1** HPLC profile of H-Gly-Pro-Arg-Gly-(Pro-Hyp-Gly)_12_-NH_2_.

HPLC gradient: 10−40% CH_3_CN in 0.05% TFA over 30 minutes at 60 °C.

**
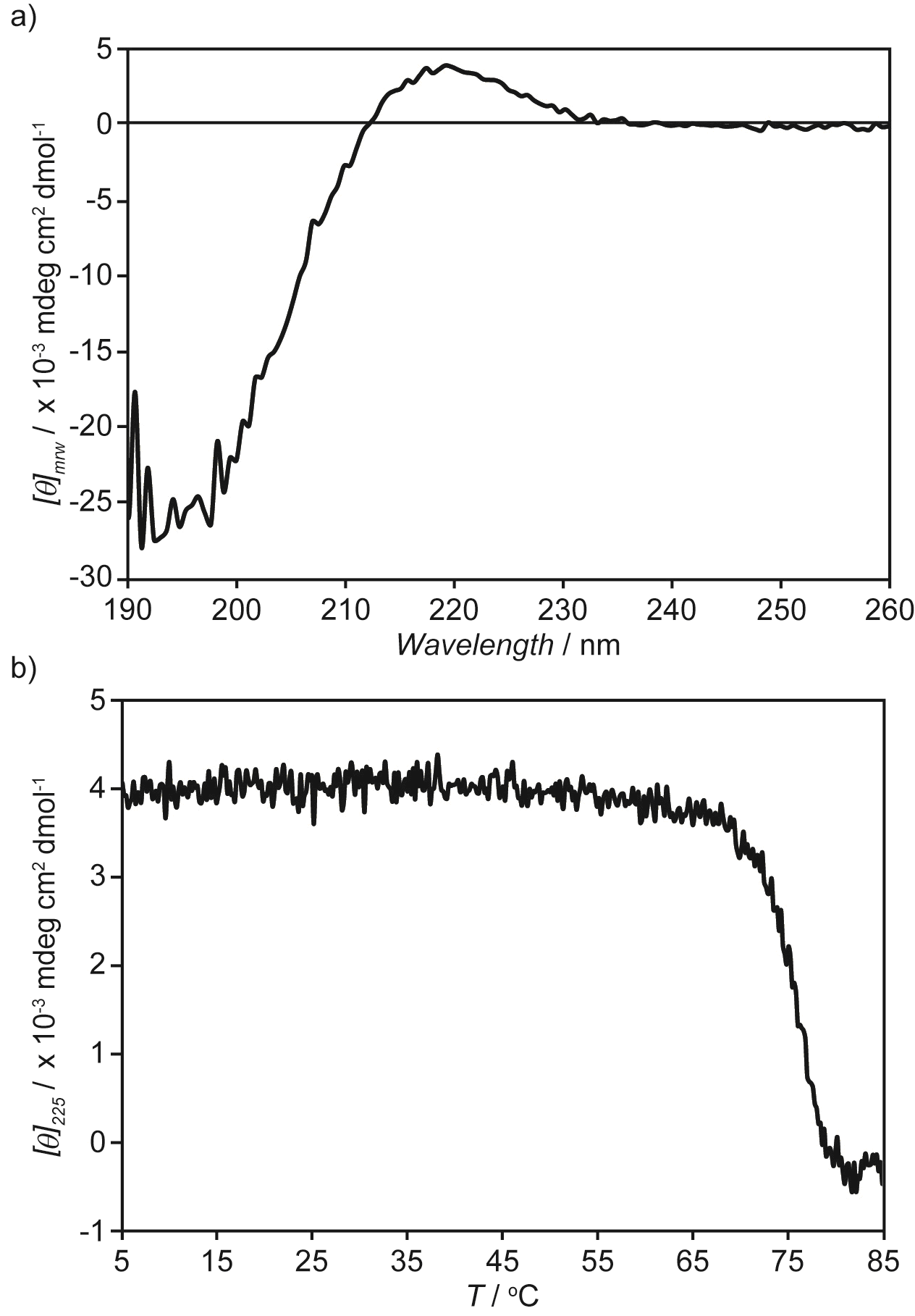
**

**Supplementary Fig. 2 CD profiles of H-Gly-Pro-Arg-Gly-(Pro-Hyp-Gly)_12_-NH_2_ in H_2_O.**

a) CD spectrum recorded at 4°C. b) Thermal melting curves of triple helices.
